# Downstream funding success of early career researchers for resubmitted versus new applications: A matched cohort

**DOI:** 10.1371/journal.pone.0257559

**Published:** 2021-11-18

**Authors:** Jamie Mihoko Doyle, Michael T. Baiocchi, Michaela Kiernan

**Affiliations:** 1 Division of Clinical Innovation, National Center for Advancing Translational Sciences, National Institutes of Health, Bethesda, MD, United States of America; 2 Department of Epidemiology and Population Health, Stanford University School of Medicine, Palo Alto, CA, United States of America; 3 Department of Medicine, Stanford University School of Medicine, Palo Alto, CA, United States of America; Northwestern University, UNITED STATES

## Abstract

**Background:**

Early career researchers face a hypercompetitive funding environment. To help identify effective intervention strategies for early career researchers, we examined whether first-time NIH R01 applicants who resubmitted their original, unfunded R01 application were more successful at obtaining any R01 funding within 3 and 5 years than original, unfunded applicants who submitted new NIH applications, and we examined whether underrepresented minority (URM) applicants differentially benefited from resubmission. Our observational study is consistent with an NIH working group’s recommendations to develop interventions to encourage resubmission.

**Methods and findings:**

First-time applicants with US medical school academic faculty appointments who submitted an unfunded R01 application between 2000–2014 yielded 4,789 discussed and 7,019 not discussed applications. We then created comparable groups of first-time R01 applicants (resubmitted original R01 application or submitted new NIH applications) using optimal full matching that included applicant and application characteristics. Primary and subgroup analyses used generalized mixed models with obtaining any NIH R01 funding within 3 and 5 years as the two outcomes. A gamma sensitivity analysis was performed. URM applicants represented 11% and 12% of discussed and not discussed applications, respectively.

First-time R01 applicants resubmitting their original, unfunded R01 application were more successful obtaining R01 funding within 3 and 5 years than applicants submitting new applications—for both discussed and not discussed applications: discussed within 3 years (OR 4.17 [95 CI 3.53, 4.93]) and 5 years (3.33 [2.82–3.92]); and not discussed within 3 years (2.81 [2.52, 3.13]) and 5 years (2.47 [2.22–2.74]). URM applicants additionally benefited within 5 years for not discussed applications.

**Conclusions:**

Encouraging early career researchers applying as faculty at a school of medicine to resubmit R01 applications is a promising potential modifiable factor and intervention strategy. First-time R01 applicants who resubmitted their original, unfunded R01 application had log-odds of obtaining downstream R01 funding within 3 and 5 years 2–4 times higher than applicants who did not resubmit their original application and submitted new NIH applications instead. Findings held for both discussed and not discussed applications.

## Introduction

Early career researchers face a hypercompetitive grant funding environment while transitioning from their academic and/or clinical training to research independence [1]. The average age of first major research grant (R01) from the National Institutes of Health (NIH) has markedly increased from 30 to 40 years of age in 1980 to 48 years of age in 2007 [2]. Furthermore, only 10% of all NIH-funded researchers hold 40% of NIH research dollars [3], reflecting a funding environment that may be in a state of “stasis”, with well-funded researchers remaining in the top funding ranks with little mobility in between [4]. Such stasis threatens the vitality and long-term sustainability of the biomedical research workforce.

Identifying effective intervention strategies that support the grant application success of early career researchers is critical as this persistent, hypercompetitive funding environment may force talented, early career researchers to pursue other occupations and deprive others of funding necessary to pursue important scientific questions. In 2017, the NIH Advisory Committee to the Director Working Group on Diversity Report recommended that intervention strategies be developed for closing the funding gaps for early career researchers [5], including intervention strategies that improve rates of resubmission among discussed, but unfunded first-time submissions [6].

Over half (51%) of principal investigators (PIs) on average across all career stages do not resubmit initially unfunded R01 applications despite published NIH data showing that grant applications resubmitted the first time have higher success rates than original submissions [7–9]. Well-controlled published studies examining whether resubmission can benefit early career PIs are limited. Although one study found that PIs who resubmitted initially unfunded applications were more likely to be funded after controlling for confounders, the study’s regression analysis included PIs from all career stages, and only those who resubmitted discussed applications that were scored [9]. Thus, it is unknown whether early career PIs, in particular, should resubmit discussed applications with unfavorable scores and should resubmit applications that were not discussed.

Studies are also mixed on whether an intervention strategy centered on resubmission would help narrow the R01 funding gap between underrepresented minorities (URM) and other well-represented groups. Ginther et al. (2011) reported that URMs were less likely than white investigators to receive R01 funding [5]. The authors concluded that since black/African American and Hispanic applicants were less likely to resubmit a revised application, providing resubmission assistance to URM PIs may help diversify the R01 pool. Conversely, more recently, Hoppe et al. (2019) reported that black/African American applicants were not less likely than white PIs to resubmit their application and criticized the Ginther et al. (2011) study for not including the influence of impact score on resubmission [9]. Yet, and as pointed out by a Hoppe et al. (2019) co-author, it is difficult to draw definitive conclusions from the Hoppe et al. (2019) analysis as it did not account for institute/center (IC) award rates as a confounder [10]. Thus, whether resubmission would differentially benefit URMs, even for applications that were not discussed and while accounting for IC award rates, is unknown.

The current observational study will provide initial empirical evidence to inform the development of intervention strategies encouraging resubmission for early career faculty at U.S. medical schools. The current study has two objectives. For the first objective, we examined the impact of resubmission on obtaining R01 funding among first-time NIH R01 applicants from medical schools. Specifically, we examined whether first-time NIH R01 applicants who resubmitted their original, unfunded R01 application were subsequently more successful at obtaining downstream R01 funding of any kind within 3 and 5 years than first-time NIH R01 applicants who did not resubmit their original, unfunded applications and instead submitted a new NIH application of any kind. For this objective, we also stratified by whether the original, unfunded R01 applications had been either discussed or not discussed, as these applications may differ by applicant and/or application characteristics. Since the focus was on early career faculty applying for R01s for the first time, we excluded faculty members with tenure. Tenured faculty are likely to have different dynamics in place (e.g., lab already established) when considering funding choices. For the second objective, we went beyond previous studies by examining whether first-time NIH R01 applicants from URMs may differentially benefit from resubmission given persistent disparities in NIH grant funding by historically underrepresented groups [5].

Here, we implemented a three-step causal inference approach designed to strengthen conclusions given decisions to resubmit R01 applications are not random (i.e., applications with favorable scores are likely to be resubmitted). First, we prepared the data set using nonparametric preprocessing [11] via optimal full matching, an analytical design that flexibly constructs sets to ensure covariate balance between those who resubmit and those who did not. This approach has been previously shown to mitigate bias while improving precision in observational studies relative to other matching techniques such as pair matching using propensity scores [11,12]. Second, as our inference step, we used generalized mixed models to estimate interaction effects while also accounting for the clustering of observations within sets created through optimal full matching. Third, because this is an observational study, we assessed the potential impact of omitted baseline-covariate differences by performing sensitivity analyses to systematically gauge the degree of influence that any omitted variables would need to attain to challenge the statistical conclusions [13].

Importantly, this study leveraged a comprehensive data set comprised of covariates previously unavailable to prior studies. We integrated two independent restricted-use population-based data sets to comprehensively assess two sets of covariates: applicant characteristics (e.g., sex, prior K Award, institutional level of NIH funding) and application characteristics (e.g., percentiles, scores, Early Stage Investigator (ESI) status, and success rate of the administrative IC). Extensive analyses of NIH funding outcomes have been limited since access to both personally identifiable information, such as age, gender, and race/ethnicity, as well as the subsequent status of unfunded grant applications are not publicly available.

If resubmission was shown to be a successful intervention strategy, then effective, upstream intervention strategies could be designed to improve resubmission efforts (e.g., proposal writing training, understanding reviewer feedback, strategies for adapting to reviewer feedback).

## Materials and methods

### Data sources

The two main data sources included the restricted-use NIH Information for Management, Planning, Analysis and Coordination database (IMPAC II) database containing information on funded and unfunded NIH grant applications and the Association of American Medical Colleges (AAMC) Faculty Roster file [14]. We also incorporated data from NIH RePORTER.

#### Population

A flow diagram for the analytical cohort sample is available in S1 Appendix. To examine first-time R01 applicants who submitted their first NIH R01 application during 2004–2014 (N = 27,545), there were five exclusions: applicants who received prior R01-equivalent awards (DP2, R23, R29, R37, and RF1) or submitted an amendment as their first R01 application; did not have a US medical school faculty appointment at the time of submission; were missing or had an unknown academic degree or race/ethnicity; had an academic title other than assistant professor (e.g., associate or full) or already had tenure; and had their first R01 application funded the first time or did not submit any additional NIH applications after the original unfunded application. U.S. medical schools roughly receive half of all NIH funding ($14.2 out of $26.9 billion) [15] and it is important to note that these results cannot be generalizable to the entire biomedical research workforce. After exclusions, there were 11,808 applicants in the analytical sample.

#### Outcomes

There were two primary outcomes: whether first-time applicants had any funded R01 applications (i) within 3 years and (ii) within 5 years of their original, unfunded R01 application. As NIH grant policy states that PIs must resubmit unfunded applications within 37 months of the new or revised application it follows [16], the 3-year window provided enough time to examine whether the original, unfunded R01 application was funded while minimizing the number of applications removed due to right censoring (e.g., applications initiated in more recent years such that there were not enough follow up years to analyze). The 5-year window spanned the typical tenure period for new assistant professors, and provided enough time to examine whether subsequent, entirely new NIH applications were funded.

The secondary and descriptive outcome was whether first-time R01 applicants were eventually awarded their original, unfunded R01 application.

#### Definition of resubmitted original R01 application versus submitted new NIH applications

We stratified by whether the first-time R01 applicants’ original, unfunded R01 applications were ***discussed*** (N = 4,789) or ***not discussed*** (N = 7,019). Then, using optimal full matching (details below), we created two comparable groups of first-time R01 applicants: (i) those who ***resubmitted their original R01 application*** and (ii) those who did not resubmit their original, unfunded R01 application and instead ***submitted a new NIH application of any kind***. If first-time R01 applicants submitted multiple NIH applications, one of which was a resubmission of their original, unfunded R01 application, these applicants were defined as having resubmitted. To identify the two groups, we isolated the grant activity code (i.e., R01), the two-letter administrative institute/center code, the six-digit serial number, and searched whether this “grant stem” serial number appeared in later application records in IMPAC II.

#### Description of covariates

*NIH Information for Management*, *Planning*, *Analysis and Coordination database (IMPAC II)*. A description of 20 key covariates used for the analysis is shown in Table 1. Including resubmission, a total of 21 covariates were used for this study. Other variables from IMPAC II used for this analysis include whether the proposed study includes human subjects and/or animal subjects, whether the application was reviewed as an Early Stage Investigator (ESI) application, whether the application was in response to a specific funding solicitation, whether the application involved multiple principal investigators, and the fiscal year when the initial, unfunded R01 application was submitted. Since prior K-awardees have been found to have higher probability of research funding success than non-K awardees [17], whether an individual was a prior K-award recipient was also included in the analysis. We also included a variable that captures the number of times a principal investigator served on an NIH study section prior to submitting their application.

**Table 1:** Descriptions of variables used for the study, Association of American Medical Colleges Faculty Roster File (AAMC), National Institutes of Health administrative data (IMPAC II), and NIH RePORT.

Variables	Description	AAMC	IMPAC II	NIH RePORT
**Applicant**				
Race/ethnicity	Race/ethnicity categorized as underrepresented minority (URM), non-Hispanic Asian, and non-Hispanic white. To ensure reporting cell sizes > 11, applicants other than Asian and white were categorized as URM, including self-identified Hispanic. Race/ethnicity from AAMC, supplemented by IMPAC II if missing.	X	X	
Sex	Sex from AAMC, supplemented by IMPAC II if missing.	X	X	
Age	Age at first R01 application.	X	X	
Degree type	Terminal degree completed (e.g., PhD, MD, MD/PhD).	X		
Prior K-awardee	An indicator of whether the applicant was a prior K-awardee.		X	
Cumulative non-R01 NIH funding	Cumulative amount of non-R01, NIH research project grant funding at the time of first R01 application.		X	
Cumulative NIH peer review service	Cumulative number of times applicants served on NIH peer review panels.		X	
Cumulative publications	Cumulative number of peer reviewed publications.		X	
Academic rank	Academic rank at the time of first R01 application; Assistant Professor vs. Other.	X		
Tenure track	Tenure track status at the time of first R01 application.	X		
Appointment subunit	Academic department: Internal Medicine, Pediatrics, Psychiatry, and Other.	X		
Institutional NIH funding tier, high	The total amount of NIH funding that the applicant’s institution was awarded in 2007 (the middle year of this matched cohort study, 2000–2014), and institutions above the 90th percentile (≥ $17,562,010) were classified as “high.”		X	
**Application**				
Percentile rank	Percentile rank of the application. Discussed applications only.		X	
Priority score	Priority (or “impact”) score of the application. NIH scoring system for priority scores changed in 2009 (old system: priority scores <100, new system: priority scores >100). Discussed applications only.		X	
Early stage investigator (ESI)	Whether application was reviewed as ESI application. The ESI policy was established November 2008 ^a^. Since this cohort spans the period of 2000–2014, a large percentage of the cohort applied before the ESI policy was established.		X	
Fiscal year	Fiscal year of the original, unfunded R01 application		X	
Success rate, application admin IC	R01 success rate of the administrative institute/center of the original, unfunded R01 application. Since no success rates were reported for the National Library of Medicine from 2004–2006, we imputed the mean for these years (17.1).			X
Solicited application	An indicator of whether the application was responding to a specific funding solicitation.		X	
Multiple principal investigators (PIs)	An indicator of whether the application involved multiple PIs. Since the policy was established November 2006 ^b^, a large percentage of the cohort applied before multi-PI policy was established.		X	
Animal subjects or human subjects	Separate indicator variables on whether human and/or animal subjects were included in the application.		X	

^a^ https://grants.nih.gov/policy/early-investigators/history.htm.

^b^ https://grants.nih.gov/grants/guide/notice-files/NOT-OD-07-017.html.

The most prognostically important variable we include in our analysis is the percentile rank/priority score of an application. Applications with more favorable priority scores/percentiles will tend to be resubmitted. After an application is submitted, but before a peer review meeting takes place, each reviewer submits preliminary scores for the applications they are assigned. Based on these preliminary scores, half of the lowest scoring applications within a study section meeting will be selected to not be discussed during the meeting. These applications are also not assigned percentile ranks or final priority scores. Among applications that are discussed a peer review meeting, an overall score is assigned to the application based an average of each reviewer’s score of the application [18]. To help account for differences in scoring behaviors across study sections, percentile ranks are assigned based on scores from the same study section during the last three meetings. Applications with lower priority scores and lower percentile ranks fared better during peer review than those with higher priority scores and percentile ranks. Details about percentile ranks can be found here: https://www.niaid.nih.gov/grants-contracts/understand-paylines-percentiles.

Data on publications comes from the NIH publications system, SPIRES, which is accessible through IMPAC II. SPIRES links publications to individuals through awards listed in the acknowledgments section of publications in PubMed and from publications that are submitted to NIH in compliance with the NIH Public Access Policy. We include the number publications prior to the first R01 application in the analysis. Given the source of these data, individuals supported on prior NIH training grants and other NIH awards may have artificially higher numbers of publications than those from unfunded or non-federal funded projects since data on publications come from an NIH system.

*American Association of Medical Colleges Faculty Roster file (AAMC)*. The AAMC has maintained a database of faculty positions at accredited U.S. medical schools since 1966 [14]. Data reported from the faculty roster file are comparable to data reported to the Liaison Committee on Medical Education, the accrediting body for allopathic medical schools in the United States [14]. Not only does this data contain detailed information on academic appointments, but the AAMC also maintains person-level information. Variables used exclusively from the AAMC database include: Date of birth, degree type (PhD, MD/PhD, and MD), academic rank, tenure track status, and appointment subunit. Age was calculated by taking the difference between the year of birth and the fiscal year of the first unfunded R01 application. Mean imputation for age (42.4) was conducted for observations missing date of birth, which affected 1569 observations in the dataset. Appointment subunit refers to the department classification of the academic appointment. For example, pediatrics and internal medicine are appointment subunit categories. Appointment subunit was categorized into four categories: Internal Medicine, Pediatrics, Psychiatry, and Other.

The advantage of using the AAMC data are two-fold. First, IMPAC II does not capture any information on tenure track status systematically and collects position information that is available in the biosketch. Tenure track status may also be important confounder since grant expectations may be different for those on the tenure track versus those who are not. The AAMC also harmonizes faculty position information (ex. Assistant Professor, Associate Professor, etc.), which circumvents the need to manually clean information based on titles on the biosketch. Second, medical school faculty may fundamentally be different from non-medical school faculty given that grant expectations for those relying on “soft money” may be different than those relying on “hard money.”

*AAMC and IMPAC II*. Data on race/ethnicity and sex were primarily taken from the AAMC but were supplemented using data from IMPAC II in cases where data were missing. In the AAMC, 11 PIs were missing data on sex and 1130 were missing data on race/ethnicity. Supplementing the demographic data in AAMC with data in IMPAC II data reduced the number of missing observations on sex to zero and reduced missing data on race/ethnicity to 155 cases (130 after prior exclusions). Using both the AAMC and IMPAC II demographic data resulted in significant decreases in missing data on race/ethnicity. Data reported by NIH from fiscal years 2016–2020 shows that 9% of data on race/ethnicity is missing or withheld in IMPAC II [19]. However, by leveraging two data sources, our study reduced the missing data on race/ethnicity to only around one percent.

Using these restricted-use data requires protecting personally identifiable information, including race/ethnicity. To ensure that cell sizes for reporting were greater than 11, racial/ethnic categories were collapsed. Individuals who self-identified as Asian or white are included as separate racial categories for the analysis. All other racial/ethnic groups are collapsed into an Underrepresented Minority (URM) category, which also includes those who self-identified as Hispanic. Cases with missing data on race/ethnicity after supplementing demographic information from IMPACII were excluded.

*NIH RePORT*. Institutional NIH funding tier was based on the total amount of NIH funding the institution associated (in 2007) with the PI’s academic appointment, which is the mid-year for this cohort (2000–2014). Institutions were binned into percentiles according to their NIH total period amount in funding. Those above the 90th percentile, or greater than or equal to $17562010, were classified as “high”. R01 success rates by IC and fiscal year were taken from the publicly available NIH RePORT website: https://report.nih.gov/success_rates/Success_ByActivity.cfm. Since no success rates were reported for the National Library of Medicine from 2004–2006, we imputed the mean for these years (17.06). The imputation affected seven observations in our dataset.

#### Descriptive results

To assess alignment with previous research, we reported the descriptive rates for obtaining R01 downstream funding for different subgroups (e.g., Discussed vs. Not Discussed and Resubmit vs. New Application). However, given these rates are not controlled or adjusted sufficiently to address causal questions, covariate imbalances need to be addressed by a careful matched design, as described below.

#### Optimal full matching (Step 1: Nonparametric preprocessing)

We used optimal full matching: (i) to improve the comparability of first-time applicants who resubmitted their original, unfunded R01 application and first-time applicants who submitted new NIH applications; (ii) to increase the inclusion of observations through flexible set sizes, which results in fewer unmatched observations and higher effect estimation precision; and (iii) to enforce within-set balance (particularly with the URM status) which has been shown to protect against inflated claims about subgroup heterogeneity [20].

Details of match quality assessment and implementation are reported in S2 Appendix. We defined match quality using standardized mean differences (SMDs), with abs(SMD)<0.20 judged as acceptable balance and abs(SMD)< 0.10 being excellent balance [21,22].

#### Regression models (Step 2: Inference accounting for study design)

Matched sets were analyzed using generalized mixed models with a logit-link [23]. There were two models for each primary outcome, stratified by discussed and not discussed applications. The first model included one fixed effect categorical variable for resubmission and a random effect for set membership. The second model included three fixed effect categorical variables for resubmission, race/ethnicity, and the interaction term for resubmission and race/ethnicity, and a random effect for set membership. Coefficients for the fixed effects terms for these models were reported. Models were performed using the “lme4” package in R [24].

#### Gamma sensitivity (Step 3: Formal sensitivity analysis for confounding)

We performed a formal sensitivity analysis using the gamma sensitivity parameter and the Cochran-Mantel-Haenszel test [25]. For interpretation, a gamma sensitivity parameter of 2 suggests the matched cohort is resilient to potential confounding. To nullify the observed differences between the groups, there would need to be a set of nearly perfect predictors of success that were unobserved (with conditionally independent variation) sufficient to increase the odds of resubmission by a factor of 2 for future successful applications [26]. Sensitivity analyses were performed using “Sensitivityfull” package in R [27].

## Results

### Descriptive results

Among discussed applications, 81% (N = 3,891 of 4,789) of first-time applicants resubmitted their original, unfunded R01 application. Among not discussed applications, only 41% (N = 2,885 of 7,019) of first-time applicants resubmitted their original, unfunded R01 application.

#### Primary outcomes (descriptive)

Among discussed applications, first-time R01 applicants who resubmitted were much more likely to obtain downstream R01 funding within 3 and 5 years than those who submitted new NIH applications. Among not discussed applications, a similar, though smaller benefit for downstream R01 funding was seen for first-time R01 applicants who resubmitted than those who submitted new applications (p<0.001).

#### Secondary outcome (descriptive)

Among discussed applications, only about half (53.8%) of first-time applicants were eventually awarded their original, unfunded R01 application. Among not discussed applications, less than a third (27.3%) were awarded their original applications.

#### Application and application characteristics

For discussed and not discussed applications, Table 2 displays unadjusted estimates for all 21 applicant and application characteristics for those who resubmitted or submitted new applications.

**Table 2:** Applicant and application characteristics of first-time R01 applicants, unadjusted covariates (2000–2014).

	DISCUSSED (N = 4789)		NOT DISCUSSED (N = 7019)	
	Priority scores and percentiles on original R01 application		No priority scores and percentiles on original R01 application	
	Resubmitted original R01 application	Submitted new NIH^a^ application	Resubmitted original R01 application	Submitted new NIH application
Sample size N (%)	3891 (81.2)	898 (18.8)	2885 (41.1)	4134 (58.9)
**Applicant characteristics** ^**b**^				
Race/ethnicity (%)				
Underrepresented minority (URM)	417 (10.7)	86 (9.6)	330 (11.4)	478 (11.6)
Non-Hispanic Asian	945 (24.3)	215 (23.9)	727 (25.2)	1117 (27.0)
Non-Hispanic white	2529 (65.0)	597 (66.5)	1828 (63.4)	2539 (61.4)
Women (%)	1361 (35.0)	312 (34.7)	1025 (35.5)	1503 (36.4)
Age	40.2 (4.6)	40.4 (4.6)	40.7 (4.7)	41.4 (5.1)
Degree type (%)				
MD	860 (22.1)	225 (25.1)	562 (19.5)	955 (23.1)
MD/PhD	614 (15.8)	157 (17.5)	450 (15.6)	609 (14.7)
PhD	2417 (62.1)	516 (57.5)	1873 (64.9)	2570 (62.2)
Prior NIH K awardee (%)	909 (23.4)	178 (19.8)	654 (22.7)	690 (16.7)
Cumulative non-R01 NIH funding	$132,590 ($302,359)	$104,384 ($285,844)	$73,964 ($202,589)	$78,438 ($257,635)
Cumulative NIH peer review service	0.2 (0.7)	0.2 (0.9)	0.1 (0.6)	0.2 (0.7)
Cumulative number of publications	5.5 (25.6)	4.4 (21.1)	4.1 (28.8)	3.3 (15.8)
Academic rank, assistant professor	3587 (92.2)	804 (89.5)	2646 (91.7)	3739 (90.4)
Tenure track status				
Tenure track	2165 (55.6)	434 (48.3)	1498 (51.9)	1780 (43.1)
Not on tenure track	1460 (37.5)	393 (43.8)	1197 (41.5)	2010 (48.6)
Tenure track missing	266 (6.8)	71 (7.9)	190 (6.6)	344 (8.3)
Appointment subunit				
Internal medicine	995 (25.6)	255 (28.4)	618 (21.4)	1000 (24.2)
Pediatrics	309 (7.9)	76 (8.5)	218 (7.6)	343 (8.3)
Psychiatry	297 (7.6)	53 (5.9)	239 (8.3)	309 (7.5)
Other	2290 (58.9)	514 (57.2)	1810 (62.7)	2482 (60.0)
Institutional NIH funding tier, high (%)	3478 (89.4)	781 (87.0)	2507 (86.9)	3531 (85.4)
**Application characteristics**				
Percentile rank (%)				
[5.6, 33.2)	1107 (28.5)	91 (10.1)	NA	NA
[33.2, 42.1)	1075 (27.6)	141 (15.7)	NA	NA
[42.1, 50.1)	898 (23.1)	285 (31.7)	NA	NA
[50.1, 93.0]	811 (20.8)	381 (42.4)	NA	NA
Priority score (%)^c^				
[20,39)	450 (11.6)	42 (4.7)	NA	NA
[39,46)	455 (11.7)	79 (8.8)	NA	NA
[46,52)	320 (8.2)	129 (14.4)	NA	NA
[52,84]	274 (7.0)	208 (23.2)	NA	NA
[131,204)	665 (17.1)	45 (5.0)	NA	NA
[204,231)	644 (16.6)	74 (8.2)	NA	NA
[231,259)	585 (15.0)	125 (13.9)	NA	NA
[259,480]	498 (12.8)	196 (21.8)	NA	NA
Early stage investigator (ESI) (%)	1117 (28.7)	316 (35.2)	496 (17.2)	1118 (27.0)
Fiscal year	2007.3 (4.2)	2007.8 (4.7)	2006.7 (3.6)	2007.7 (4.2)
Success rate of application admin IC	19.0 (4.6)	18.6 (5.1)	19.67 (4.3)	18.45 (4.8)
Solicited application (%)	1053 (27.1)	233 (25.9)	860 (29.8)	1011 (24.5)
Multiple principal investigators (%)	105 (2.7)	33 (3.7)	58 (2.0)	228 (5.5)
Application included human subjects (%)	1567 (40.3)	394 (43.9)	1022 (35.4)	1768 (42.8)
Application included animal subjects (%)	2131 (54.8)	445 (49.6)	1630 (56.5)	2139 (51.7)

^a^ Abbreviations: Appl., Application. IC, NIH Institute/Center. Cumul., Cumulative. SD, Standard Deviation. NIH, National Institutes of Health. NA, Not Applicable. Admin, Administrative.

^b^ Means and standard deviations are reported unless otherwise indicated. Column percentages are reported. Cumulative non-R01 funding includes smaller NIH research project grants such as R03 and R21 awards.

^c^ NIH scoring system for priority scores changed in 2009 (old system: Priority scores <100, new system: Priority scores >100). Of discussed, original, unfunded R01 applications, 39% (N = 1,499) of the resubmitted R01 applications and 51% (N = 458) of the new, submitted NIH applications were reviewed with the new scoring system.

### Optimal full matching (Step 1: Nonparametric preprocessing)

Table 2 shows the unadjusted applicant and application characteristics of first-time R01 applicants, and Fig 2 shows the standardized mean differences (SMDs) in covariates between those who resubmitted vs. those who submitted a new application both before and after optimal full matching stratified by those applications that were discussed vs. those that were not discussed.

**Fig 1:**
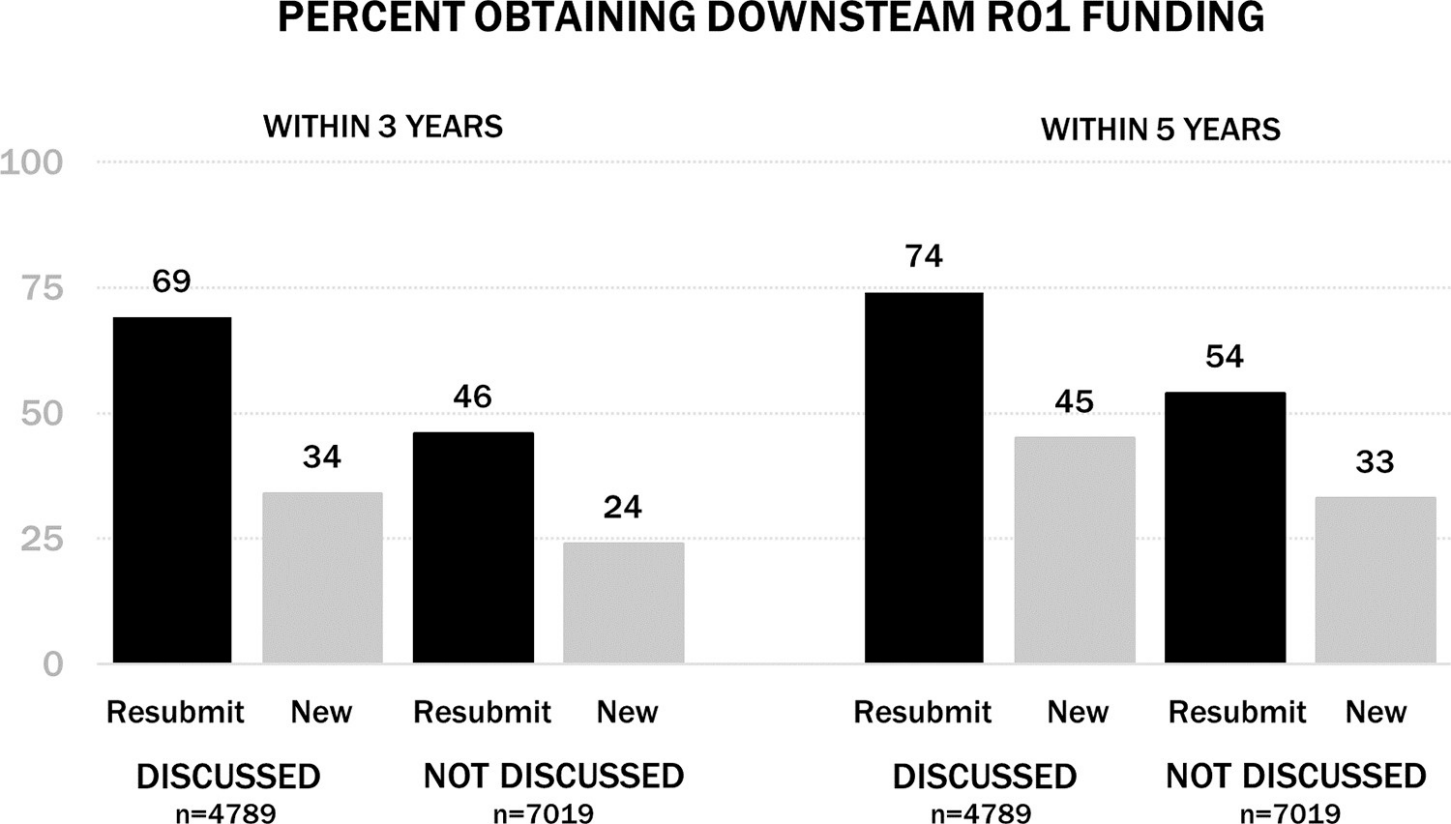
Distribution of obtaining R01 downstream funding, unadjusted.

Table 2 shows significant differences in priority scores and percentiles of applications that were resubmitted versus those that were not and underscores the importance of not drawing conclusions based on award rates observed in Fig 1. The two groups began as incomparable. Thus, the optimal full matching design is used to reduce these baseline imbalances.

Among discussed applications, prior to matching, first-time R01 applicants who resubmitted differed from those who submitted new NIH applications on two of the 21 applicant and application characteristics, priority scores and percentile ranks, as imbalances were outside a SMD of absolute value of 0.2. After matching, all characteristics were within a SMD of absolute value of 0.2, and most achieved a SMD of less of absolute value of 0.1.

Among not discussed applications, prior to matching, first-time R01 applicants who resubmitted differed from those who submitted new NIH applications on three of the 18 characteristics (priority scores and percentile rank were not applicable for not discussed applications) as imbalances were outside a SMD of absolute value of 0.2. First-time applicants who resubmitted were less likely to submit as an Early Stage Investigator, submitted in an earlier fiscal year, and applied to an IC with a higher success rate than those who submitted new applications. After matching, all characteristics were within a SMD of absolute value of 0.2, and most achieved a standard mean difference of less of absolute value of 0.1.

**Fig 2:**
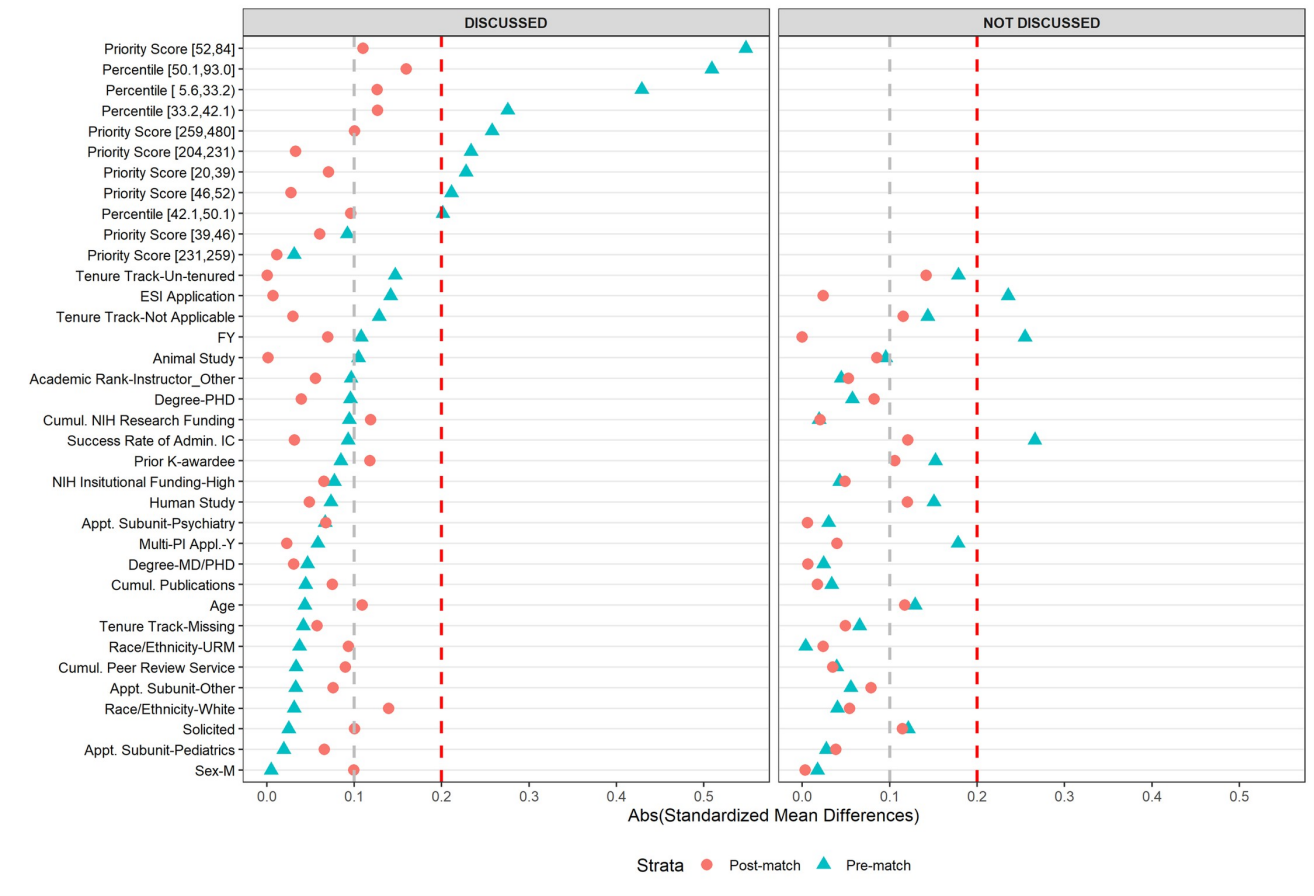
Love plots showing absolute standardized mean differences pre- and post- optimal full matching.

Thus, for both discussed and not discussed applications, optimal full matching minimized baseline covariate differences, improving the plausibility of unbiased estimation for the regression models. Fig 2 displays the Love plots showing standardized differences before and after optimal full matching.

### Regression models (Step 2: Inference accounting for study design)

#### Main effect of resubmission

Results from the regression models are shown in Table 3. Among discussed applications, first-time applicants who resubmitted had a log-odds of obtaining any downstream R01 funding that was 4.17 times higher within 3 years and 3.33 times higher within 5 years than those who submitted new NIH applications.

**Table 3:** Regression models testing main and interaction effects for the two primary outcomes by discussed and triaged original, unfunded applications.

	DISCUSSED						NOT DISCUSSED					
	Model 1 Main effects only			Model 2 Includes interaction effect			Model 1 Main effects only			Model 2 Includes interaction effect		
Primary outcomes	Coeff	*P* value	OR (95% CI)	Coeff	*P* value	OR (95% CI)	Coeff	*P* value	OR (95% CI)	Coeff.	*P* value	OR (95% CI)
**Within 3 years**												
Intercept	-0.71	<0.001	0.49 (0.42–0.57)	-0.71	<0.001	0.49 (0.41–0.59)	-1.21	<0.001	0.30 (0.27–0.32)	-1.18	<0.001	0.31 (0.28–0.34)
Resubmit [Ref = Submitted new appl.]	**1.43** ^a^	**<0.001**	**4.17 (3.53–4.93)**	**1.42**	**<0.001**	**4.13 (3.37–5.07)**	**1.03**	**<0.001**	**2.81 (2.52–3.13)**	**1.04**	**<0.001**	**2.83 (2.47–3.25)**
Race/ethnicity [Ref = Non-Hispanic white]												
Asian				0.13	0.47	1.14 (0.80–1.62)				-0.0004	0.99	1.00 (0.84–1.19)
URM^b^				-0.48	0.09	0.62 (0.36–1.08)				**-0.34**	**0.01**	**0.71 (0.55–0.93)**
Resubmit x race/ ethnicity												
Resubmit x Asian				-0.17	0.40	0.85 (0.57–1.25)				-0.10	0.41	0.90 (0.70–1.16)
Resubmit x URM				0.55	0.07	1.73 (0.95–3.15)				0.18	0.33	1.19 (0.84–1.70)
**Within 5 years**												
Intercept	-0.24	<0.001	0.79 (0.68–0.91)	-0.21	0.03	0.81 (0.68–0.98)	-0.74	<0.001	0.48 (0.44–0.51)	-0.71	<0.001	0.49 (0.45–0.54)
Resubmit [Ref = Did not resubmit]	**1.20**	**<0.001**	**3.33 (2.82–3.92)**	**1.15**	**<0.001**	**3.15 (2.57 3.84)**	**0.90**	**<0.001**	**2.47 (2.22–2.74)**	**0.89**	**<0.001**	**2.44 (2.14–2.78)**
Race/ethnicity [Ref = Non-Hispanic white]												
Asian				-0.03	0.88	0.97 (0.69–1.38)				0.02	0.85	1.02 (0.86–1.19)
URM				-0.31	0.24	0.74 (0.44–1.23)				**-0.37**	**0.002**	**0.69 (0.55–0.88)**
Resubmit x race/ ethnicity												
Resubmit x Asian				0.05	0.79	1.05 (0.72–1.55)				-0.11	0.36	0.90 (0.70–1.14)
Resubmit x URM				0.44	0.13	1.54 (0.88–2.71)				**0.35**	**0.04**	**1.42 (1.02–2.00)**

^a^ **Boldface** indicates statistical significance and reference categories are indicated in brackets ([])

^b^ Abbreviations: URM, Underrepresented minority.

Among not discussed applications, first-time applicants who resubmitted also experienced benefits. First-time applicants who resubmitted their original, unfunded R01 applications had a log-odds of obtaining any R01 downstream funding that was 2.81 times higher within 3 years and 2.47 times higher within 5 years than those who submitted new NIH applications.

#### Interaction of race/ethnicity with resubmission

Among discussed applications, the point estimates for the interaction term were in the positive direction for the models for both 3 years and 5 years, suggesting an increased benefit of resubmission for URM applicants, but neither point estimate was statistically significant. Among not discussed applications, the point estimates for the interaction term were positive for the models for both 3 years and 5 years, but only the model for 5 years was statistically significant.

### Gamma sensitivity (Step 3: Formal sensitivity analysis for confounding)

Among discussed applications, the gamma sensitivity parameter was 2.81 for the within 3 years analysis and 2.29 for the within 5 years analysis. Among not discussed applications, the sensitivity parameter was 2.39 for the within 3 years analysis and 2.14 for the within 5 years analysis.

## Discussion

Encouraging early career researchers to resubmit R01 applications is a promising potential modifiable factor and basis for an intervention strategy. First-time R01 applicants who resubmitted their original, unfunded R01 application had log-odds of obtaining downstream R01 funding of any kind within 3 and 5 years 2–4 times higher than applicants who did not resubmit their original application and submitted new NIH applications instead (ranging from OR 2.47 [CI 2.22–2.74] to OR 4.17 [CI 3.53–4.93]). These findings held for both discussed and not discussed applications. First-time applicants who never applied for any NIH funding after their original, unfunded R01 application were explicitly excluded *a priori* from the analytical sample. Although estimated effects were large even after matching on 21 applicant and application characteristics, we urge caution accepting these results uncritically or as if results came from a randomized controlled trial to avoid a naïve conclusion that all applications should be resubmitted.

How might resubmitting unfunded R01 applications lead to higher log-odds of downstream R01 funding for first-time applicants—especially as only 54% of discussed applicants and 27% of not discussed applicants were eventually awarded their original, unfunded R01 application? First, resubmission may strengthen the persistence or grit of first-time applicants [28]. In randomized experiments in education [29], interventions emphasizing high standards and beliefs that students were capable of meeting standards [30,31], increased students’ likelihood of submitting essay revisions and improved the quality of their final drafts, including among URM students.

Second, resubmission could cultivate new, necessary skills for handling reviewer critiques, both substantively and psychologically, which could improve future NIH applications. As one “How to” guide published by *Science* stated, “You might say that having your proposal rejected gets you that much closer to getting a grant if you handle rejection and use it to your advantage” [32]. In recent descriptive reports [33–35], academic institutions now implement educational intervention programs to support early career researchers while also rigorously vetting their applications by peers and senior mentors to avoid common mistakes, such as hastily prepared applications, and to ensure essential conceptual and methodological details are included. Third, resubmission of unfunded R01 applications may keep first-time applicants oriented to securing R01 funding rather than switching to other types of NIH funding or other funding sources. Little is known about how early career researchers could best time and combine submissions for different types of NIH mechanisms and is an area for future research.

We found mixed evidence that resubmitting R01 applications may differentially benefit URM first-time applicants. Point estimates for the interaction term were in the positive direction for all four models, and one model returned a statistically significant coefficient. However, given the analytical sample was a near-census of all applicants with a low percentage of URM first-time applicants that reflected the actual NIH R01 pool, statistical power was likely quite low for detecting interaction terms with highly unbalanced subgroups and thus large standard errors. In addition, due to small samples, we could not examine URM subgroups separately, so lack of differential benefit could be due to population heterogeneity [5]. More broadly, many URM faculty navigate challenges that limit time to work on grant applications such as inadequate mentoring, lack of institutional support, and other social, cultural, and environmental factors [5,36–38]. There is also documented evidence that URM faculty are often asked to participate in time-consuming, service-related activities to promote diversity [38] and spend more time than non-URM faculty mentoring students [39].

This study had several limitations. First, decisions to resubmit unfunded applications or to submit new applications may be based on information unavailable and uncorrelated with any of the 21 covariates, such as conversations with program officers or mentors who counsel not to resubmit even though applications received strong priority scores or counsel to resubmit despite applications being not discussed and could bias estimates toward resubmission. Yet, the gammas from the sensitivity analyses were greater than 2, meaning unobserved variables would need to be nearly perfect predictors of success unaccounted for by the observed covariates, and more than double the odds of resubmission. Second, this sample focused on US medical school faculty submitting their first R01 application and cannot be generalized to the entire NIH-funded biomedical research workforce or to investigators outside of the United States. Third, this analysis does not include prior history of foundation or industry funding, which may influence the probability of an application being discussed or funded [40,41]. Finally, some applicants may have used the feedback on their initial review to scale back and refine their original R01 application into small grant mechanisms (i.e., R21, R03, etc.).

The current findings are timely given the increasing average age to the first R01 award and future of the biomedical research workforce. This study also provides insight about the potential effectiveness of proposed intervention strategies focused on application resubmission, such as those recommended by the NIH Advisory Committee to the Director Working Group on Diversity. Pursuing such intervention strategies may be useful for early career researchers, particularly those from URM groups. This study quantified the benefits to resubmitting initially unfunded applications by US medical school faculty using a matched design that integrated valuable, restricted-use data from NIH and the AAMC, which allowed the study to better capture early career researchers and their tenure track status. It remains essential to support and sustain a robust pipeline of researchers who can address important scientific questions to meet critical public health needs.

## Supporting information

S1 Appendix Flow diagram describing the arrival of the final sample. (PDF)

S2 Appendix Match quality assessment and implementation. (PDF)

S1 Dataset Minimum dataset for balance plots. (CSV)

S2 Dataset Minimum dataset for discussed applications, matched sample. (CSV)

S3 Dataset Minimum dataset for not discussed applications, matched sample. (CSV)

S4 Dataset Minimum dataset for gamma sensitivity test, discussed applications. (CSV)

S5 Dataset Minimum dataset for gamma sensitivity test, not discussed applications. (CSV)
