# Supplementary material for: Downstream funding success of early career researchers for resubmitted versus new applications: A matched cohort
Source: PLoS One. 2021 Nov 18;16(11):e0257559. doi: 10.1371/journal.pone.0257559 (PMC8601543; doi:10.1371/journal.pone.0257559)
Supplement: S1 Appendix — (PDF) [file pone.0257559.s001.pdf]

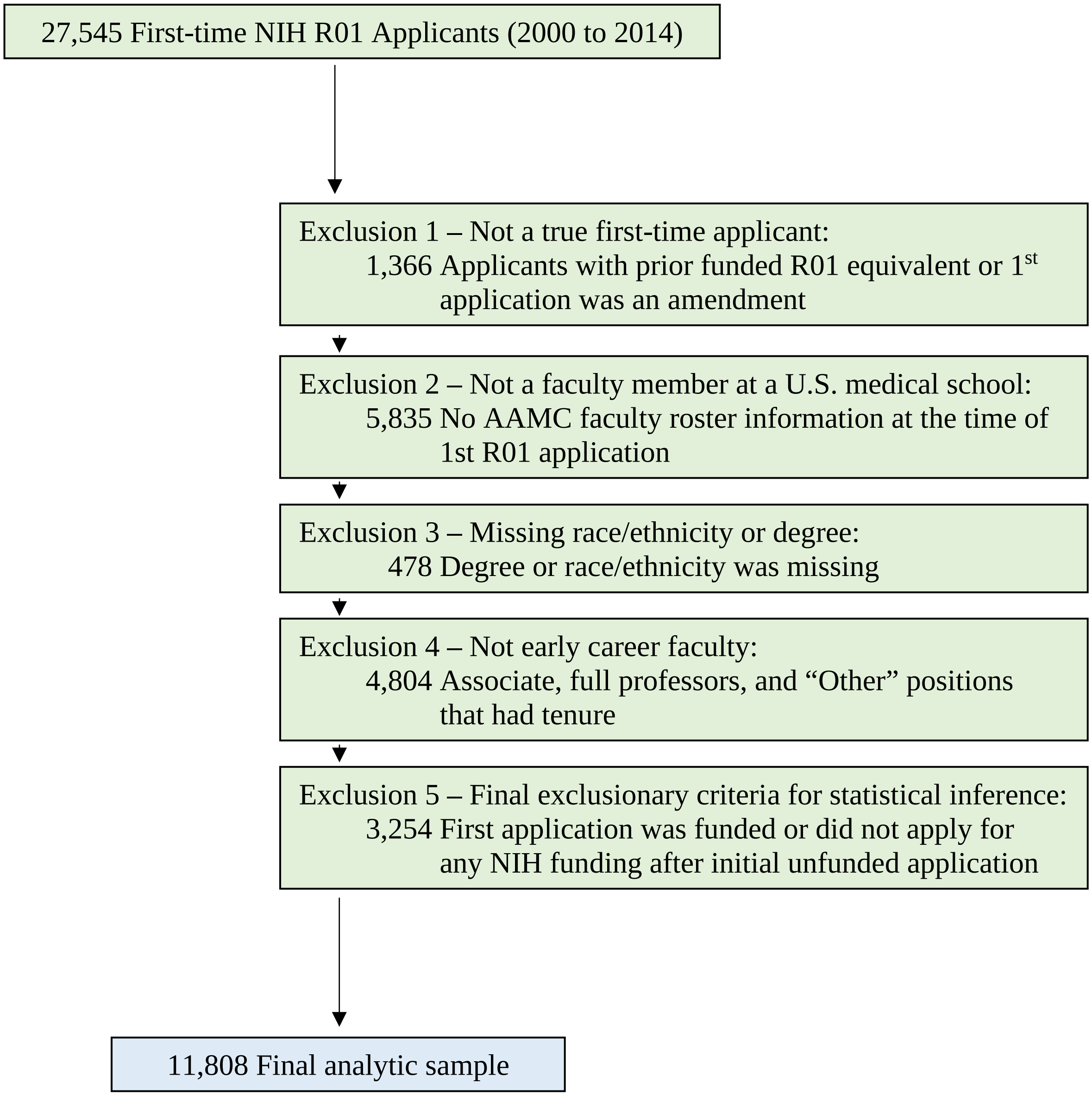
27,545 First-time NIH R01 Applicants (2000 to 2014)
Exclusion 1 – Not a true first-time applicant:
1,366 Applicants with prior funded R01 equivalent or 1st application was an amendment
Exclusion 2 – Not a faculty member at a U.S. medical school:
5,835 No AAMC faculty roster information at the time of 1st R01 application
Exclusion 3 – Missing race/ethnicity or degree:
478 Degree or race/ethnicity was missing
Exclusion 4 – Not early career faculty:
4,804 Associate, full professors, and "Other" positions that had tenure
Exclusion 5 – Final exclusionary criteria for statistical inference:
3,254 First application was funded or did not apply for any NIH funding after initial unfunded application
11,808 Final analytic sample
