# Supplementary material for: Downstream funding success of early career researchers for resubmitted versus new applications: A matched cohort
Source: PLoS One. 2021 Nov 18;16(11):e0257559. doi: 10.1371/journal.pone.0257559 (PMC8601543; doi:10.1371/journal.pone.0257559)
Supplement: S2 Appendix — (PDF) [file pone.0257559.s002.pdf]

Set specific weights were then used to assess the outcome. For both discussed and triaged applications, optimal full matching was implemented using Mahalanobis distance. For discussed applications, we used exact matching for the pre- vs post-2009 period since NIH rescaled their priority scores in 2009.[16] For triaged applications, we also used exact matching within fiscal year. We defined match quality using standardized mean differences (SMDs), with abs(SMD)<0.20 judged as acceptable balance and abs(SMD)< 0.10 being excellent balance,[17,18] and then compared the match quality for alternative matching protocols including propensity score-based distances. The best match quality (the smallest SMDs for the most important covariates) was achieved with optimal full matching and was performed using the "Optmatch" package in R.[19]
